# Assessing the ownership, usage and knowledge of Insecticide Treated Nets (ITNs) in Malaria Prevention in the Hohoe Municipality, Ghana

**DOI:** 10.11604/pamj.2017.28.67.9934

**Published:** 2017-09-22

**Authors:** Kunche Delali Nyavor, Margaret Kweku, Isaac Agbemafle, Wisdom Takramah, Ishmael Norman, Elvis Tarkang, Fred Binka

**Affiliations:** 1 Faculty of Education, University of Ottawa, Canada; 2 School of Public Health, University of Health and Allied Sciences, Ho, Volta region, Ghana; 3 Institute for Security, Disaster and Emergency Studies, Sandpiper Place, Langma, Central Region, Ghana; 4 HIV/AIDS Prevention Research Network, Cameroon, Kumba, South-West region, Cameroon

**Keywords:** Malaria prevention, children, ITN ownership, ITN usage, knowledge, caregivers

## Abstract

**Introduction:**

Malaria remains one of the top five killer diseases in sub-Saharan Africa (SSA) and its burden is skewed towards pregnant women and children under five. Insecticide Treated Bed-Net (ITN) usage is considered one of the most cost-effective, preventive interventions against malaria. This study sought to assess ownership, usage, effectiveness, knowledge, access and availability of ITNs among mothers with children under five in the Hohoe municipality.

**Methods:**

In August 2010 a cross-sectional survey was carried out in 30 communities, selected using the WHO 30 cluster sampling technique. In the selected communities, mothers/caregivers with children under five years were selected using the snowball method. Data were collected through questionnaires and direct observation of ITN. Descriptive statistics was used to analyse the data collected.

**Results:**

A total of 450 mothers/caregivers were interviewed and their mean age was 30 ± 7 years. ITN ownership was 81.3%, and usage was 66.4%. The majority (97.8%) of the mothers/caregivers said ITNs were effective for malaria prevention. Awareness about ITNs was high (98.7%) and the majority (52.9%) had heard about ITNs from Reproductive and Child Health (RCH) Clinic and antenatal care ANC clinic (33.6%). Over 60% of the ITNs were acquired through free distribution at RCH clinics, clinic and home distribution during mass immunization sessions. The majority of the mothers/caregivers (78.6%) knew the signs and symptoms of malaria, what causes malaria (82.2%) and who is most at risk (90%).

**Conclusion:**

Behaviour change communication strategies on ITN use may need to be further targeted to ensure full use of available ITNs.

## Introduction

Malaria is a major public health problem and one of the leading causes of maternal and child deaths particularly in Africa. Globally, 198 million cases of malaria occurred in 2013 and the disease resulted in 584,000 deaths [1]. An estimated 90% of all malaria deaths occurred in Africa and 78% of these deaths occurred in children under five [1]. A consensus by the World Health Organization (WHO) in 1992 mentioned insecticide-treated nets (ITNs) as the most promising preventive measure against malaria. ITN_s_ have been described as the best intervention that could save the lives of more children than any other single intervention apart from breast feeding and oral rehydration therapy [2, 3]. An ITN is a treated, safe net, effective in reducing human contact with mosquitoes. The distribution of ITNs has been shown to reduce malaria episodes, severe disease, and malaria-related death in endemic regions. Insecticide-treated nets have helped to reduce malaria episodes by 48-50% [4] and if universally used, could prevent an estimated 7% of global under-five mortality [2]. They are also connected with statistically significant reductions in the risk of low birth weight and foetal loss [5].

It appears that household ITN ownership declines within 2-3 years after mass campaigns [6]. A 37% and 13% decline in ITN ownership were recorded within three years of campaign without further net input in Sierra Leone and Togo respectively [7]. Ownership may not translate into usage as usage does not appear to be near-universal. Most studies report usage rates in the range of 60-80% [8–10], although World Malaria report documented 90%+ usage [11]. In Thailand, actual usage as determined by surprised home visits was 70-73% while reported usage was 85% [9], but in Tanzania, actual usage was 85% and reported usage was 97% [12]. Although survey methods could account for some of the differences in ITN usage across countries, current deployment initiatives are based on evidence of its cost effectiveness and the consistent and large benefits provided by ITNs in Africa as compared to Asia [4, 5, 10]. Statistics indicate that malaria is responsible for 9% of overall mortality in Ghana, accounts for 44% of outpatient attendance, 13% of all hospital deaths, and 22% of mortality among children under five years [13]. At an estimated cost of US$1.2 per person protected per year, ITNs are considered to be one of the most cost-effective health interventions in Ghana [14]. However, estimates from Africa indicate that only 3% of children under five years sleep under ITNs, while up to 22% of Ghanaian children under five sleep under any bednet [15]. Ghana as a signatory to the May 2006 Abuja declaration and as part of the objectives of the Roll Back Malaria (RBM) programme, Ghana was to increase ITN ownership to 80% and use to 60% by 2010; by 2015 there should be 100% ownership and 80% usage [15]. Factors such as the knowledge level of the people about ITNs, environmental and socio-cultural factors (such as perceptions and beliefs about causes of malaria, perceptions about the use of ITNs and family size), as well as sleeping arrangements, may facilitate participation or nonparticipation in ITN campaigns [16–18]. In Hohoe municipality, subsidized ITNs from the RBM programme have been distributed free of charge to all households with children under two years of age since the 2006 national immunization days. However, ITN usage with respect to ownership and level of effectiveness in the Hohoe municipality has not been evaluated. The objective of this study was to assess ITN ownership, usage and knowledge of ITNs and identify factors that hinder ITN acquisition and usage in Hohoe municipality in the Volta Region of Ghana.

## Methods

### Study design and setting

This was a cross-sectional study among mothers/caregivers with children under 5 years in the Hohoe municipality. The municipality is one of the twenty-five administrative districts in the Volta Region and it is located in the central part of the region. It is bounded to the north and northwest by Jasikan district, South by Afadjato South district, East by Republic of Togo, West by Biakoye district and Southwest by Kpando municipality. The municipality has a population of 167,743 people, with 48.1% male to 51.9% female ratio estimated from the 2010 population census [19]. The municipality covers an area of 1,403 sq. km and Hohoe, the municipal capital has a population of 63,000 people. The vegetation is of two types: the forest and semi-savannah vegetation zones. The climate is tropical with temperatures varying between 22°C and 37°C. The average annual rainfall in the municipality is 1,592 mm with approximately 1,296 mm of rain falling between April and October [20]. There are three main seasons: the major rainy season from April to August, the minor rainy season from September to November, and the dry (Harmattan) season from December to March.

### Sample size determination and sampling

A simple population proportion formula assuming a malaria prevalence of 5% in the Volta region, alpha level of 0.05 and 80% power was used to obtain a sample size of 384. This number was increased to 450 to account for 15% non-response rate. The WHO 30 cluster survey system was used to select communities for the study. This method is a commonly used two-staged cluster sampling method, thought to be sufficient for most sampling of community health factors. This study used 30 by 15 cluster sampling design which means that 30 communities in the municipality were selected at random and 15 qualified mothers/caregivers were recruited into the study in each selected community. The names of all the communities were listed to form a sampling frame of clusters. Simple random sampling was used to select the 30 communities from the 2000 population census list of communities in the municipality. The 30 communities were selected by one stage cluster sampling. The centre (important landmark) of the selected community was located and a random direction was chosen by spinning a pen. A random number between 1 and N (sample size) was chosen to represent the house that contains the first household (starting point) to be surveyed. A household was eligible if there was a child under five years in it. If there were more than one eligible households in a house, the eligible household was chosen at random. For the purpose of this study, a household was defined as a group of people who live under the same roof and eat together.

### Data collection

The study population was mothers/caregivers with children under five years, in households in the selected communities. Information on mothers/caregivers’ background characteristics was obtained using a pretested semi-structured questionnaire. Informations on the sex and age of the child, and the caregiver’s level of education, marital status and occupation were obtained from the respondents through a one-on-one interview. Also caregivers were asked questions to ascertain their knowledge about ITNs, signs and symptoms of malaria, ownership and use of ITNs, as well as the prevention of children from getting malaria.

### Statistical analysis

Data were entered twice using Epi Data software. The accuracy of data input was checked and validated using customized validation programme. After cleaning the data it was exported into STATA version 10 (Stata Corporation, Texas, USA) for analysis. The dependent variables were ITN ownership and use. Effectiveness was defined as ITNs being able to kill mosquitoes, or prevent them from biting. The independent variables were knowledge of ITNs, socio-economic factors, health service factors, availability and accessibility of ITNs. Descriptive statistics such as frequency, proportion and charts were used to describe the categorical data whilst mean and standard deviation were used to describe the quantitative variables.

### Ethical considerations

Approval was obtained from the Kwame Nkrumah University of Science and Technology (KNUST) Institutional Review Board (IRB) before the commencement of the study. Permission to carry out the study was also obtained from the Hohoe Municipal Health Directorate and verbal permission was granted by the chiefs/opinion leaders of the communities selected. Written informed consent was sought from the mothers/caregivers included in this study.

## Results

### Background characteristics of mothers/caregivers

A total of 450 mothers or caregivers with a child aged less than five years were interviewed. The mean age of the children in the survey was 20 ± 14 months. Out of the 450 children, 47.8% were males. The ages of the children ranged from 1– 59 months. The mean age of the mothers/caregivers was 30 ± 7 years ranging from 17-53 years. The majority (68.7%) of respondents had completed Junior High School (JHS) (Table 1). In terms of occupation, 38.7% of the mothers/caregivers engaged in trading, 37.6% were in farming, 13.6% were hairdressers/ dressmakers and 1.6% were professionals (Table 1). About 95% of the mothers/caregivers were Christians. With respect to marital status, 86.9% said they were married, 11.8% were separated and the remaining were divorced/ widowed. Household size ranged from 3 to 5 people. Thus, 28.2% of respondents had household size of 3 people, 27.3% had household size of 4 and 21.3% had housed size of 5.

**Table 1:** Background information of mothers/caregivers and their under five children (N=450)

Characteristics	n (%)
Age of child (months) [Mean ± SD]	20.1 ± 13.9
**Sex of child**	
Male	215 (47.8)
Female	235 ( 52.2)
Age of mothers/caregivers (years) [Mean ± SD]	29.6 ± 7.2
**Level of education**	
None	24 (5.3)
Primary	66 (14.7)
Junior High School (JHS)	309 ( 68.7)
Senior High School (SHS)	49 (10.9)
Tertiary	2 (0.4)
**Occupation**	
Unemployed	39 (8.7 )
Trading	174 (38.7)
Farming	169 (37.6)
Hairdressing/ Dressmaking	61 (13.6)
Nursing/ Teaching	7 (1.6)
**Religion**	
Christian	427 ( 94.9)
Islam	19( 4.2 )
Traditional	4(0.9)
**Marital status**	
Married	391 (86.9)
Separated	53 (11.8)
Divorced/ Widowed	6 (1.3)

### Ownership of ITN

**Figure 1:**
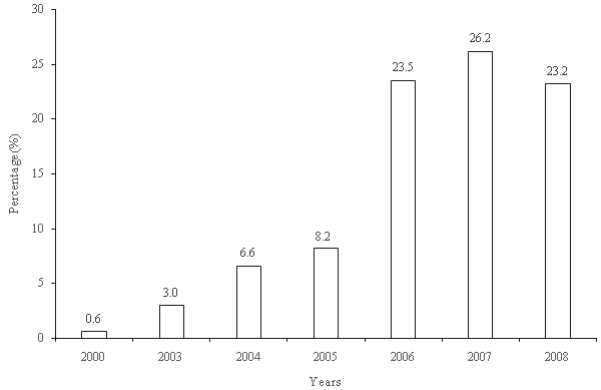
Acquisition of ITNs over the years

Of the 450 mothers/caregivers, the majority, (81.3%) said that they own an ITN in their households whilst 18.7% did not own ITNs in their households. Reasons given for not owning ITN included financial constraints (12%), not around when ITN was distributed (4.2%), no child under five years within household at the time of distribution (1.6%) and shortage of ITN in locality (0.9%). Among those who owned an ITN, 61.2% had one, 33.1% had two and the remaining had 3 or 4 (Table 2). The modes of acquisition of ITN were through the free distribution and donations at ANC clinics, Reproductive and Child Health (RCH) clinics, National Immunization Days (NIDs) programme (Polio immunization), friends and relatives. Majority (62.8%) got free ITNs whilst 37.2% purchased the ITNs (Table 2). The cost of ITN purchased ranged from a minimum of US$ 1.00 to a maximum of US$ 3.00. Sources where the ITNs were bought include: Chemical shop (31.6%), Market (28.7%), RCH clinics (24.3%) and ANC clinics (15.4%). ITNs acquisition increased gradually between 2000 to 2007 and declined from 26.2% in 2007 to 23.2% in 2008 (Figure 1).

**Table 2:** Mothers/ caregivers ITN ownership, source, use and effectiveness

Variable	n (%)
Ownership of ITN (N= 450)	
**Yes**	366 (81.3)
**No**	84 (18.7)
How was the ITN acquired(n= 366)	
**Free**	230 (62.8)
**Bought**	136 (37.2)
Sources of the free ITN (n= 230)	
**RCH clinics**	162 (70.4)
**ANC clinics**	34 (14.8)
**At home during NIDs**	34 (14.8)
Where did you buy the ITN (n=136)	
**Chemical Store**	43 (31.6)
**Market**	39 (28.7)
**RCH clinics**	33 (24.3)
**ANC clinics**	21 (15.4)
Number of ITNs owned per household (n= 366)	
**1**	224 (61.2)
**2**	121 (33.1)
**3**	16 (4.4)
**4**	5 (1.4)
UseITN last night (n=366)	
**Yes**	243 (66.4)
**No**	123 (33.6)
ITNs are effective (n=366)	
**Yes**	358 (97.8)
**No**	8 (2.2)

### Use of ITNs

Up to 34% of the mothers/caregivers did not use an ITN the previous night before the survey as shown in Table 2. The reasons why children did not sleep under the ITN the previous night included: no mosquitoes in the locality (10.0%), feeling uncomfortable (6.2%), heat causing children to cry (7.5%) and ITN was washed (3.6%). Almost all mothers/caregivers (97.8%) said ITNs were effective because mosquitoes were prevented from biting them, and about 20% said mosquitoes were seen dead on the ground every morning.

### Knowledge about malaria

**Figure 2:**
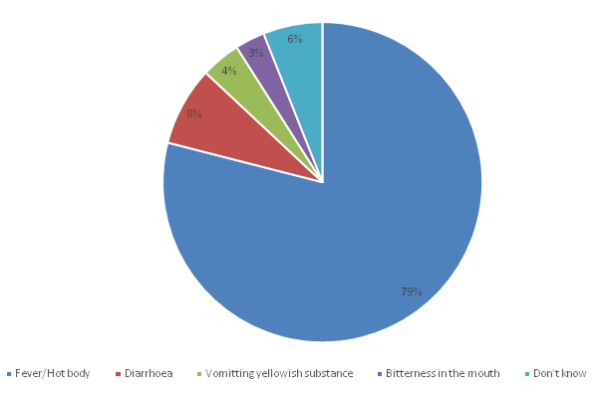
Knowledge about signs and symptoms of malaria

Almost all the mothers/caregivers have heard about ITN in the municipality. The majority, 52.9% heard about ITN from the RCH clinic; other information sources include: the ANC clinic (33.6%), community gathering (durbar) (8.6%), Radio (3.8%) and Television (1.1%). Only 1.3% of mothers/caregivers said they have never heard of ITN. The mothers/caregivers had knowledge about malaria and were able to identify the local name for malaria in their community. The local names for malaria documented from the study are “Asra” or “Ndorgbe” among the Ewe speaking communities; “Atikesi”, “Buwi”, “Evi” and “Ortoyeebe” among the Guans and “Suule” which is common among the Moslims (Zongo community). About 80% of mothers/caregivers said malaria presents itself as hot body or fever, diarrhoea (8.0%), vomiting yellowish substance (4.0%), bitterness in the mouth (3.2%), whilst (6.2%) did not express their knowledge about Malaria (Figure 2). Majority of the mothers/caregivers (82.2%) said malaria is transmitted through mosquito bites, 10.4% associated the cause of malaria to playing in the sun, 5.3% believed malaria is caused by dirty environment and 2.0% attributed it to drinking dirty water. About 90% of the mothers/caregivers were of the view that children under five years were the most vulnerable, whilst the remaining percentage were of the view that pregnant women were also at a great risk for malaria. Almost all the mothers/caregivers did something to prevent their children from getting malaria. The use of ITNs was the most common means of malaria prevention in children under five years at the household level and this method was used by 66.4% of the mothers/caregivers. Other methods of malaria prevention mentioned were, cleaning of the environment (20.7%), use of herbal preparations (12.0%), mosquito coils (4.7%), and mosquito sprays (2.7%), and the remaining 3.6% did not identify any methods currently being employed at the household level for malaria prevention.

## Discussion

Malaria occurs every year and efforts by WHO and RBM partners have promoted ITNs as a form of personal protection that reduces illness, severe disease and deaths in malaria endemic regions. In this study, ownership was defined as households that have an ITN. The study revealed that 81.3% of the surveyed households had at least one ITN and about two-thirds of those who owned an ITN used it the previous night of the survey. The 2014 Ghana Demographic and Health Survey (GDHS) report indicate a 76.1% ownership of ITN for the Volta Region [21], which is close to the estimates reported from this study. However, the 42.8% of ITN ownership reported for the Volta region in the 2008 GDHS report differed from with the estimates reported in this study [22]. The difference in ownership reported in this study as compared to estimates from the 2008 GDHS could be due to inability on the part of mothers/caregivers to differentiate between any mosquito net and an ITN. Previous studies in other African countries have reported varying proportions of ownership and this can be attributed to differences in socio-demographic characteristics and cultural beliefs about the symptoms and causations of malaria [17, 23, 24].

The outcome for ownership in Hohoe municipality is above the Abuja target of 80% ITN ownership by the end of 2010. Also, the ownership results show significant improvement in the ownership of ITN in the Hohoe municipality as compared to the national household ownership of 41.7%, as presented by the GDHS in 2008. There is even a greater improvement demonstrated by this study when the results are compared to the outcome of a survey conducted in 2004 by NetMark. In the NetMark study, it was found that the percentage of households owning at least one net was 38%, compared to 81.3% in the Hohoe Municipality as reported by this present study. The NetMark study in 2004 also found variations in the ownership of ITN between the comparator sites. In the Keta site, ownership of ITN was 64%, but fell to 19% in the Kumasi sites and 17% in the Accra sites. This indicates higher ITN ownership in different parts of the country based on the region or district of residence as environmental factors associated with Malaria varies disproportionately [17]. Also the improvements in the Hohoe municipality may be attributed to regular health education during child welfare clinics, government waving tax and free distribution of the nets through ANC clinics and national immunization days (NID) programmes to the most vulnerable groups. However, barriers such as financial constraints, not around when ITN was distributed, shortage of ITN in locality and having no child under five years in household at the time of free ITN distribution as reported by mothers/caregivers may undermine efforts at achieving 100% ownership by 2015 as enshrined in the Abuja declaration.

More importantly, ownership may not translate into usage as usage does not appear to be near-universal. Usage of ITN was defined as sleeping under ITN the previous night before the survey. About 66.4% of respondents who owned ITNs used them the previous night before the survey. The 2008 GDHS reported that 26.3% of women and 43.7% of children sleep under an ITN [22]. The variations in ITN usage between the 2008 GDHS and this present study may be due to analytical differences as this study did not segregate ITN use by mother and child. However, our results are in agreement with the 2014 GDHS which reported that 53.7% of households in the Volta Region slept under an ITN the night before the survey [21]. Studies from other African countries have reported usage rates in the range of 60-80% which is in agreement with ITN usage reported in this study [10, 25, 26]. Other studies from Africa have reported ITN usage as low as 33.5% [24] and 19.6% [27]. This represents the dynamics in ITN usage across Africa, indicating the successes attained by different countries in the fight against Malaria. These studies from various African countries have reported varying proportions of ownership and usage which have been attributed to differences in socio-demographic characteristics and cultural beliefs about the symptoms and causations of malaria [17, 23, 24]. Thus only two-thirds of households who owned an ITN used it the previous night before the study. Reasons for non-use of ITNs as reported in this study included perceived absence of mosquitoes and perceived discomfort and generation of heat by ITNs. A similar study in Ethiopia identified low awareness on malaria prevention, undermining the extent of malaria, unavailability of separate sleeping room, poor condition of ITNs and unavailability of enough ITNs to the household members as the main reasons for non-use [24]. A previous study in the Eastern and Central regions of Ghana demonstrated that caregivers´ beliefs about symptoms, causation and groups most vulnerable to malaria were significantly associated with ITN usage [17].

### Awareness of ITNs

In 2000, ITNs were just being introduced to the public. Ten years down the line, majority of the respondents (98.7%) in this study have heard about ITNs in the Hohoe municipality and sources of information were mainly from RCH clinics and ANC clinics. A series of surveys conducted in 2000 and 2004 in Nigeria, Senegal, Uganda and Zambia indicated that awareness of ITNs was nearly universal in all the countries but Nigeria [28]. The dynamics of ITN awareness in Nigeria varies from State to State with as high as 93% in the Southern States and as low as 36% in the Northern States [29, 30]. Although the respondent’s awareness on malaria prevention was near 100%, it was not translated into ITN use, which is consistent with previous studies in Ghana [13,17]. Similarly, several cross-sectional studies have shown that women in some African countries have reasonably good knowledge on the cause and prevention of malaria as shown in this study. However, the extent of ITN use is not as good as their level of knowledge and awareness about ITNs in malaria prevention especially during pregnancy [31, 32]. This may be due to differences in malaria transmission intensity which varies throughout the year and inadequate access to health information in some localities [32]. As already stated, the high level of awareness on ITNs in this study might be attributed to intensification of health education through both micro (ANC, RCH and Community durbar) and the macro (Radio and TV) media. A similar study in a rural community in Southern Nigerian identified radio/Television and hospital as the main sources of information about ITN use for malaria prevention which is in consonance to the findings of this present study [33].

### Knowledge about malaria and perceived benefits of ITNs

Over thirty percent of the nets were purchased from licensed chemical shops and 28.7% were bought from the open market, creating a total of (60.3%) of the value being attributable to the commercial sector. About 24.3% of the ITNs were accessed from the RCH clinic and an additional 15.4% came from ANC. This seems to suggest that the respondents had very high level of knowledge on the availability and places where ITNs can be assessed. The findings of this study suggests the commercial sector was an important source of ITNs and a significant partner in the availability of ITNs as reported previously [28]. In April 2000 at the Roll Back Malaria (RBM) African Summit in Abuja, Nigeria, there was a consensus by heads of States of malaria-prone countries to undertake some combination of education, demand creation, reduction of taxes and tariffs on ITNs, commercial ITN market development, and programs to reach the most vulnerable populations with subsidized ITNs. Thus the cost of nets was moderate in hospitals/clinics than in commercial centres, such as chemical shops and markets. While at the hospitals/clinics the nets were sold for less than US$ 1.00, the nets cost between US$2.00 and US$3.00 at the commercial centres. The differences in price may be attributed to efforts by the Government of Ghana to subsidise ITNs for children under five (5) years and pregnant women as enshrined in the Abuja declaration on Malaria [15].

Nearly all of the respondents who use ITN consider it effective as they believe it prevents mosquito bites and some even confirmed seeing mosquitos on the ground every morning. The NetMark study in 2004 in Accra, Keta, Kumasi and Tamale concluded that most people were using ITNs as a measure for preventing malaria and avoiding mosquito bites and also killing of mosquitoes which is in agreement with the findings of this study. More than three-quarters of the respondents related malaria to hot body or fever, 20.4% believed malaria is a disease caused by mosquito bite whilst others said malaria is a sickness caused by unhygienic environment. These responses indicate high levels of knowledge about Malaria as indicated in previous studies in the Eastern and Central regions of Ghana [17]. Other signs mentioned were diarrhoea, vomiting yellowish substances, and bitterness in the mouth. The present findings are comparable to the results of a study in Accra, Ghana which report that the most commonly identified signs and symptoms of malaria among children included hot body (fever), yellow eyes and urine, vomiting and refusal to feed [34].The high knowledge associated with the cause and spread of malaria shows a high probability that a person will choose a bed-net as a means of protection. This supports the evidence that for high targets of ITN to be achieved there should be high knowledge on malaria and ITN use as an effective means of preventing mosquitoes from causing the disease [35].

### Limitations

In this survey, there could be a potential bias in measuring ITN use among the children under five years of the mother/caregiver. It was found to be less likely to observe ITN use of all the children under five years in the household as this was based on self-reports by the mother/caregiver. Again, this study did not differentiate between ITNs and long-lasting insecticide nets (LLINs). This study simply asked if the household owns an ITN, but the respondent may not be well informed about what an ITN is and whether her net qualifies as one. Hence the use of the RBM definition is important for future studies in order to give precise meaning to data concerning ITNs. The study also relied on a cross-sectional survey conducted after the main rainy season in August when mosquito density and malaria transmission is high. Thus ITN use may be more likely to be higher than during the dry season. But, it may be useful for understanding of the reasons why ITN owned households did not use it.

## Conclusion

Awareness about malaria and ITNs was high as well as ITN ownership, but its utilization was comparatively low. Further progress in ITN utilization for malaria prevention can be achieved by specifically targeting populations in malaria endemic areas through appropriate behaviour change communication strategies to ensure the correct use of available ITNs.

### What is known about this topic

- 22% of Ghanaian children under five years were sleeping under any bed-net before 2008;
- Ghana was to increase ITN ownership to at least 80% by 2010;
- Ghana was to increase ITN usage to at least 60% by 2010.

### What this study adds

- 81.3% ITN ownership compared to the 80% target by 2010;
- 66.4% ITN usage compared to the 60% target by 2010;
- 98.7% awareness about ITN.

## Competing interests

The authors declare no competing interest.
